# Utilization of Tenting Pole Abutments for the Reconstruction of Severely Resorbed Alveolar Bone: Technical Considerations and Case Series Reports

**DOI:** 10.3390/jcm13041156

**Published:** 2024-02-19

**Authors:** Dong-Seok Sohn, Albert Lui, Hyunsuk Choi

**Affiliations:** 1 Department of Dentistry and Oral and Maxillofacial Surgery, Daegu Catholic University School of Medicine, Daegu 42472, Republic of Korea; 2 Private Practice, Calgary, AB T2P 2Y3, Canada; dralbertlui@gmail.com; 3 Department of Dentistry and Prosthodontics, Daegu Catholic University School of Medicine, Daegu 42472, Republic of Korea; hschoi@cu.ac.kr

**Keywords:** vertical ridge augmentation, guided bone regeneration, bone grafts, peri-implantitis

## Abstract

Introduction: Although various surgical techniques have been utilized in the reconstruction of severely resorbed alveolar bone, its regeneration is still regarded as a major challenge. Most of the surgical techniques used in advanced ridge augmentation have the disadvantages of prolonging the patient’s edentulous healing and increasing the need for surgical revisits because simultaneous implant placement is not allowed. This report presents a new and simplified method for advanced ridge augmentation, which utilizes a vertical tenting device. Case Presentation: The first case presented the reconstruction of the mandibular posterior region with severely resorbed alveolar bone due to peri-implantitis using tenting pole abutment for ridge augmentation. The second and third cases presented three-dimensional ridge augmentations in severely resorbed ridges due to periodontitis. The last case presented horizontal ridge augmentation using a vertical tenting device. All cases were performed under local anesthesia. Implants were simultaneously placed in the bone defect area. A vertical tensioning device was then connected to the implant platform to minimize the collapse of the bone graft during the bone regeneration period due to the contraction of the soft tissue matrix. A sticky bone graft was transplanted onto the exposed surface of the implant and on top of the vertical tensioning device. After covering with an absorbable barrier membrane, the soft tissues were sutured without tension. Conclusions: In all cases, prosthetic restorations were provided to patients after a bone grafting period of 5–6 months, leading to a rapid restoration of masticatory function. Results tracked for up to 6 years revealed observed stable reconstruction of the alveolar bone. The use of a vertical tenting device can prevent the collapse of biomaterials in the augmented ridge during the healing period, leading to predictable outcomes when achieving three-dimensional ridge augmentation.

## 1. Introduction

Tooth loss not only affects a patient’s masticatory function but also has a negative impact on their overall health and can cause aesthetic problems [1]. Fixed or removable prostheses using dental implants have been widely utilized in modern dentistry to address these issues. The use of implants for the restoration of dental function is considered the gold standard because natural aesthetics, functional effectiveness, and long-term success are supported [2]. However, implant placement in the site of alveolar defects that involve vertical bone deficiency is still considered a challenging task. Bone loss after tooth extraction is indeed a significant concern, especially during the first six months post-extraction [3]. The gradual and continuous process of bone loss throughout life is attributed to the absence of dental vascularization and the lack of functionality in the periodontal ligament [4]. Following tooth extraction, bone resorption in the residual ridge is unavoidable. According to Botticelli et al.’s study, difficult tooth extraction procedures may also result in additional bone loss due to surgical trauma [5]. Socket preservation, performed simultaneously with immediate implant placement following tooth extraction, is recognized for its ability to enhance the regeneration of residual bone tissue [6]. The post-extraction socket preservation procedure using innovative demineralized autologous tooth-derived biomaterial may be a predictable procedure by which to produce new vital bone that is able to support the dental implant rehabilitation of maxilla edentulous sites [7,8]. Failure to preserve the socket with a biomaterial after tooth extraction can lead to significant bone loss and excessive resorption. Numerous surgical techniques have been described for augmentation of the severe atrophic alveolar ridge [9,10,11,12,13]. These surgical procedures have been reported to have common drawbacks, such as an increase in surgical time, increased trauma from surgery and a prolonged edentulous period for the patient, due to the implant not being placed at the same time [14]. Bone grafts applied to severely resorbed mandibles often undergo rapid resorption due to a deficiency in soft tissue volume that contract about the graft. The surgical technique involves the use of dental implants to generate a tenting effect, facilitating the consolidation and preservation of volume in bone grafts. This approach provides a dependable and enduring reconstruction of severely resorbed mandibles, minimizing complications typically associated with alternative methods [15]. This minimizes and shortens the edentulous healing period because implants are placed simultaneously [16]. This report describes four cases of three-dimensional ridge augmentation utilizing tenting pole implants and abutments to streamline the surgical procedure and reduce the healing time for partially dentate patients.

## 2. Case Presentations

**Figure 1:**
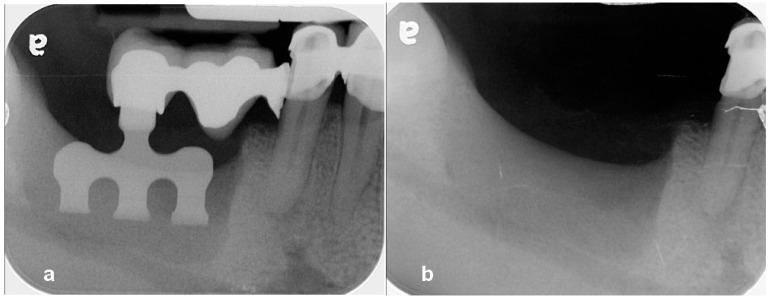
(**a**) A periapical radiograph revealed fracture restoration and severe bone resorptionaround the blade implant. (**b**) Note the severe three-dimensional bone defect after removal of the implant.

**Figure 2:**
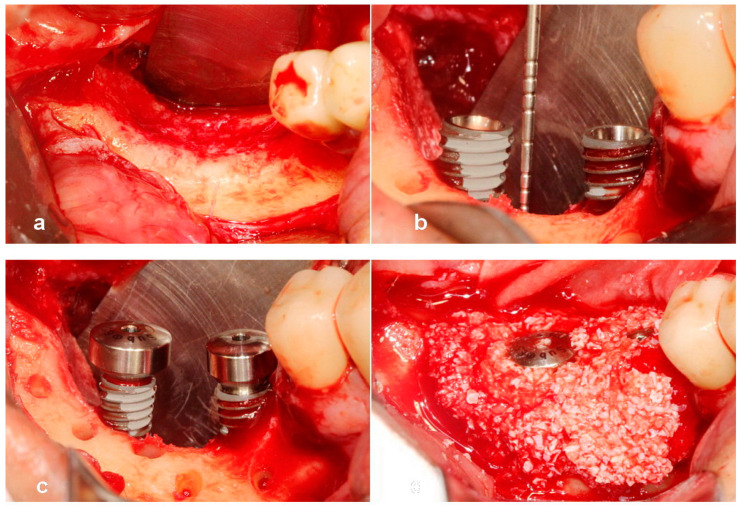
(**a**) Note the severe bony defect. (**b**) Implants were placed 2 mm subcrestally to crestal level of the adjacent proximal alveolar bone. A 6 mm high vertical defect was revealed. (**c**) A 3 mm high healing abutment was seated on each implant platform to function as a vertical tenting device. (**d**) A composite of sticky bone was grafted over the vertical defect.

**Figure 3:**
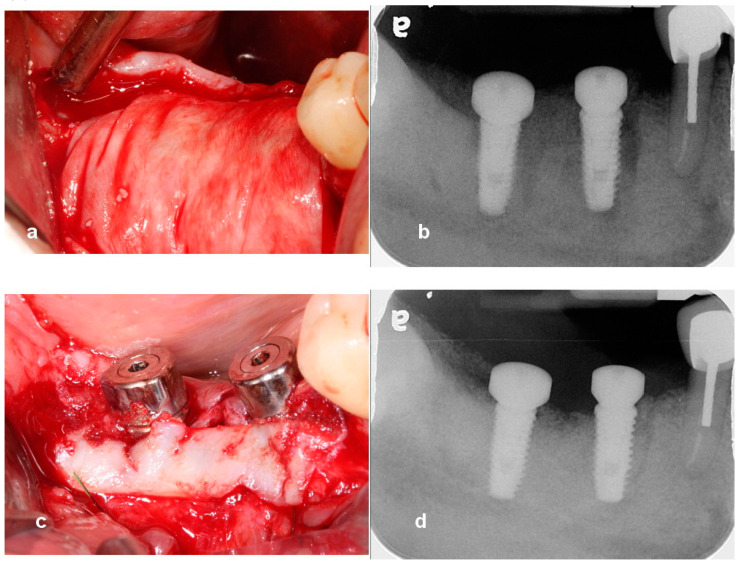
(**a**) A collagen barrier was used to cover the grafted site. Bone tacks were not used to stabilize the membrane. (**b**) A radiograph taken immediately after surgery shows bone graft around healing abutments and implants. (**c**) The uncovering was carried out after 5 months of healing. Suture-less free gingival graft was performed to attach gingiva around implant-supported restoration. (**d**) A periapical radiograph reveals successful ridge augmentation after 5 months of healing.

**Figure 4:**
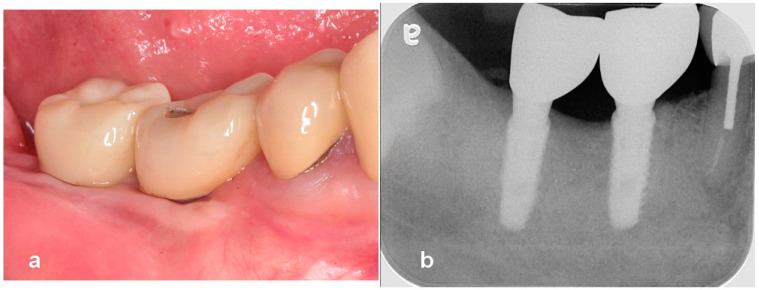
(**a**) An intraoral image after 5 years of loading. Note healthy gingiva around the definitive fixed restoration. (**b**) A radiograph showing stable marginal bone at 5 years of function.

**Case 1.** A 65-year-old man presented with a complaint of masticatory difficulty on the right posterior mandible. A restoration supported by a blade implant, which was connected to premolars, had fractured, and exhibited mobility. This blade implant was removed with forceps under local anesthesia, and soft tissue healing was allowed for six weeks. A pre-operative radiograph indicated severe horizontal and vertical bone deficiency in the edentulous ridge (Figure 1). The surgical procedure was performed under local anesthesia after the IV administration of preoperative antibiotics (Flomoxef, Flumarin^®^, Ildong Pharm, Seoul, Republic of Korea). The patient’s venous blood was taken from the forearm to create autologous fibrin glue and a concentrated growth factor membrane to prepare sticky bone, as first described by Sohn et al. [17]. A crestal incision was made through the periosteum to the bone and the retromolar pad, as well as anterior and posterior vertical incisions connecting to the crestal incision, which were made beyond the mucogingival junction to mucosa at a 45-degree angle. The lingual flap was coronally and lingually released with a periosteal elevator to dissect the periosteum and superficial fibers of the mylohyoid muscle. The periosteum of the buccal flap was released with a no. 15c blade. Any soft tissue on the bony defect was completely removed with a bony scraper tip connected with piezoelectric bone surgery (Surgybone; Silfradent srl, Sofia, Italy). To determine the occlusion and the orientation of implant placement, A surgical guide (BonePen guide, Acrodent Co., Kimhae, Republic of Korea) was employed to prepare the sites of the first molar and the second molar implant according to the manufacturer’s instruction. Under-osteotomy using a drill that was 1 mm narrower than the implant diameter was applied to obtain the initial stability of the implant. Two 4 mm wide × 10 mm long implants (Dentis implant, Daegu, Republic of Korea) were placed in the edentulous ridge as tenting pole screws. Implant platforms were placed 2 mm subcrestally to the adjacent proximal bone height. Approximately 6 mm of the implants was left exposed. A 3 mm high healing abutment was placed on the platform of each implant to function as a vertical tenting device. Small decortications were made with a round bur on the buccal cortex. A mixture of 1 g of bovine bone (InterOss^®^, Sigmagraft Biometerials, Fullerton, CA, USA) and 1 cc of allograft (Allo-Bone^®^, CG Bio Co., Seoul, Republic of Korea) was mixed with autologous fibrin glue to create a sticky bone graft. This composite sticky biomaterial was then grafted onto the defect. A resorbable collagen barrier (Remaix, Matricel GmbH, Herzogenrath, Germany) covered the bone graft. Membrane tacks or membrane stabilization sutures were not used. A tension-free suture was applied (Figure 2). The uncovering of implants was performed after 5 months of healing. A suture-less free gingival graft was simultaneously performed to provide the attached keratinized gingiva to the implant-supported restoration. A superficial horizontal incision was made at the muco-gingival junction and over-extended to adjacent teeth with a no.15 blade. Two superficial vertical incisions were made at the end of the horizontal incision and extended to the base of the vestibule. Muscular tissue was dissected on the recipient site. The apically repositioned flap was stabilized at the base of vestibule with two periosteal sutures using a synthetic absorbable surgical suture (coated vicryl, Ethicon LLC, Guaynabo, PR, USA). The dimensions of the recipient bed were measured with a periodontal probe. After packing a wet gauze moisturized with normal saline on the recipient bed, a thin (>1 mm thick) gingival graft was harvested to minimize bleeding from the donor site and the gingival graft was placed on the recipient bed. To stabilize it, a few drops of N-butyl-2-cyanoacrylate (Histoacryl, B.BRAUN Surgical, S.A, Carretera de Terrassa, Spain) were applied immediately along the superior margin of the graft, and immediately dried with a gentle air blow from a three-way air syringe. Periodontal dressing (COE-PAK^TM^, GC, Alsip, IL, USA) was applied to protect the wound site and to provide compression to the free gingival graft during the initial healing period. Suture and periodontal dressing were removed after 7 days. A definitive restoration was delivered after a 1 month loading of a provisional restoration. After 5 years of loading, there was favorable maintenance of the augmented ridge. (Figure 2, Figure 3 and Figure 4).

**Figure 5:**
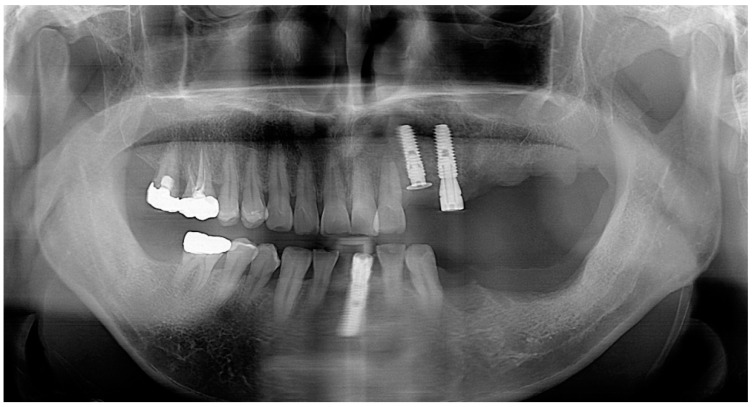
Note severe vertical defect on the left mandibular edentulous ridge.

**Figure 6:**
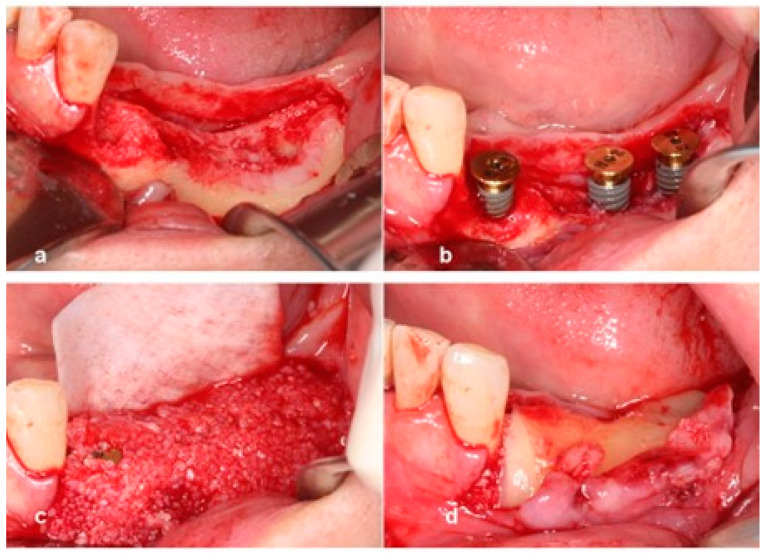
Intraoral image of surgical procedures. (**a**) A severe three-dimensional defect was revealed after releasing buccal and lingual flaps. (**b**) Implants were placed 2 mm subcrestally to the adjacent proximal bone height. Approximately 6 mm of exposure of the implant were seen. SANTA was placed on the implant as a vertical tenting device to function as a vertical space maintainer and was tightened to 10 Ncm. (**c**) A sticky autologous osteoinductive tooth bone was grafted on the defect, and a collagen barrier covered the bone graft. Membrane tacks were not used to stabilize the collagen barrier. (**d**) Tension free suture using 40 nylon was performed.

**Figure 7:**
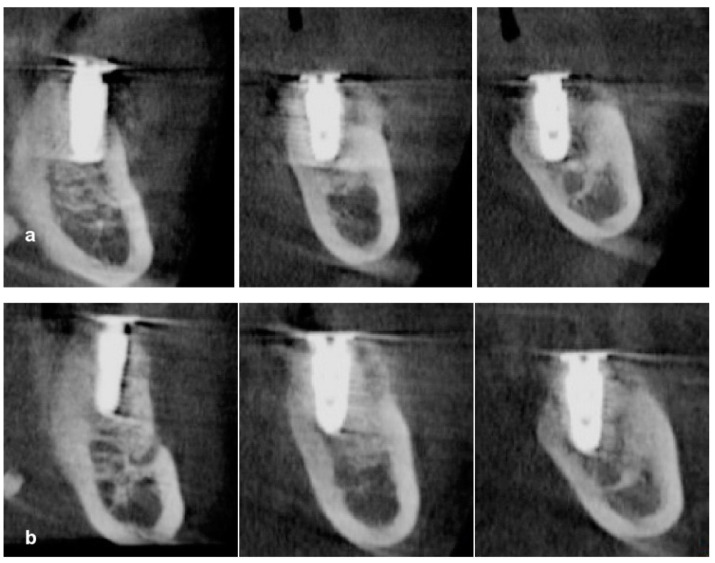
(**a**) CBCT scans immediately after surgery. (**b**) CBCT scans after 5 months of healing reveal successful ridge augmentation on bony defect and over implant platforms.

**Figure 8:**
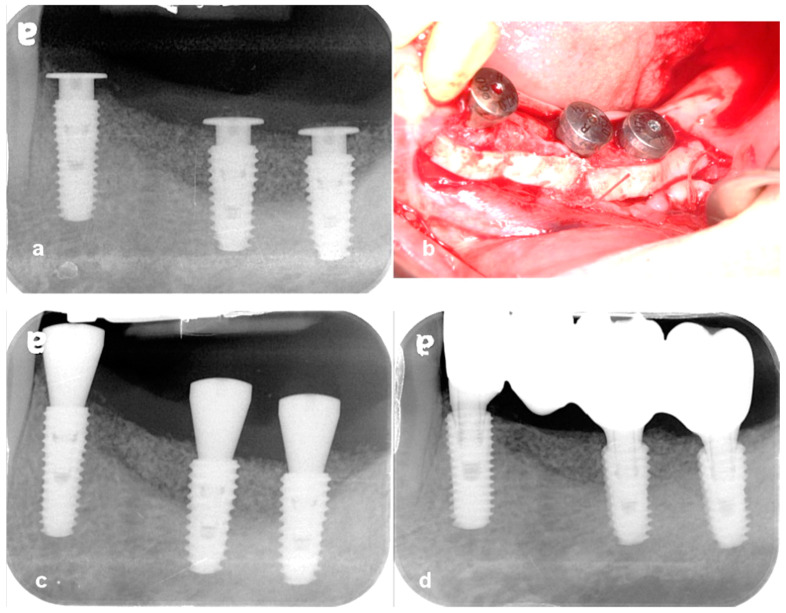
(**a**) A postoperative periapical radiograph reveals bone grafting on the defect. (**b**) The uncovering was performed after 22 weeks of healing. A suture-less free gingival graft was utilized to obtain attached gingiva. (**c**) A periapical radiograph reveals stable ridge augmentation over implant platform. (**d**) A periapical radiograph after the delivery of a final restoration.

**Figure 9:**
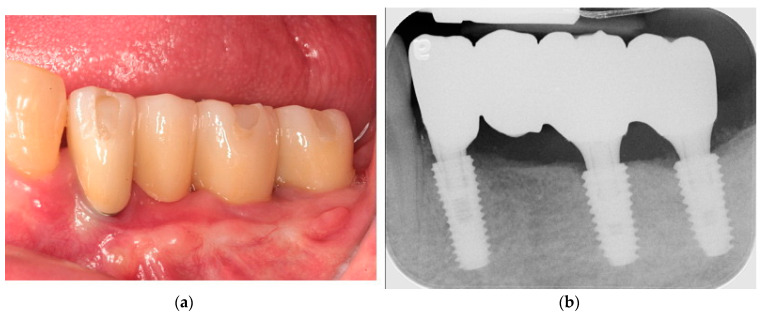
(**a**) An intraoral image after 2 years of loading. (**b**) A periapical radiograph indicating stable bone graft over the implant platform after 2 years of loading.

**Case 2.** A 53-year-old man presented with severe mobility of upper left maxillary posterior teeth, lower left premolars, and lower central incisors. Hopeless teeth were extracted, and implants were placed immediately on the sites of the upper right canine, first molar, and lower left central incisor. He visited our department again to receive an implant-supported restoration in the edentulous left posterior mandible and maxilla after 6 weeks of healing. The extracted teeth were prepared for decalcified osteoinductive particulate tooth bone grafts using a vacuum-ultrasonic machine (VacuaSonic system, CosmoBioMedicare, Seoul, Republic of Korea) 2 h before surgery, and a tooth bone graft was prepared for sticky bone, as described in case report 1. Preoperative radiograph indicated severe three-dimensional bone defects (Figure 5). The surgical procedure was performed under local anesthesia (2% lidocaine with epinephrine 1:100,000) after the intravenous injection of antibiotics (Flomoxef, Flumarin^®^, Ildong Pharm, Seoul, Republic of Korea). Severe vertical and horizontal defects were revealed after the elevation of a full thickness mucoperiosteal flap. A lingual flap was also released with a periosteal elevator. Under-osteotomy was performed at the first premolar and the first and second molar sites using a drill that was 1 mm narrower than the implant diameter in order to achieve initial implant stability. Implants that were 4.1 mm wide × 11.5 mm long and 5 mm wide × 10 mm long (Biotem Implant, Busan, Republic of Korea) were placed at the first premolar and molar areas, respectively, with favorable stability and as an alternative to tenting pole screw, in order to act as a vertical tenting device. Implant platforms were placed 2 mm subcrestally to the adjacent proximal bone height. A vertical tenting pole abutment (SANTA^®^, Biotem Implant Co., Busan, Republic of Korea) with a 2 mm cuff height was placed on the implant platform to maintain the volume of the sticky tooth bone graft during the healing period. Then, the 5–6 mm exposure of implants was revealed. Sticky tooth bone was grafted on to the bony defects with the purpose of maintaining space. A collagen barrier membrane (Lysoguide, Oscotec Co., Seoul, Republic of Korea) was used to cover the bone graft, and two concentrated growth factor membranes were placed over the collagen barrier to accelerate wound healing. Tension-free primary sutures were applied. Healing was uneventful until the second surgery. The uncovering was performed after 22 weeks of healing. A plain radiograph and cone beam computed tomogram (CBCT) scan images revealed stable ridge augmentation. To widen the attached keratinized gingiva, a suture-less free gingival graft, as described in case report 1, was performed. A final zirconia-based restoration was delivered after 6 weeks loading of the progressive restoration (Figure 6, Figure 7, Figure 8 and Figure 9).

**Figure 10:**
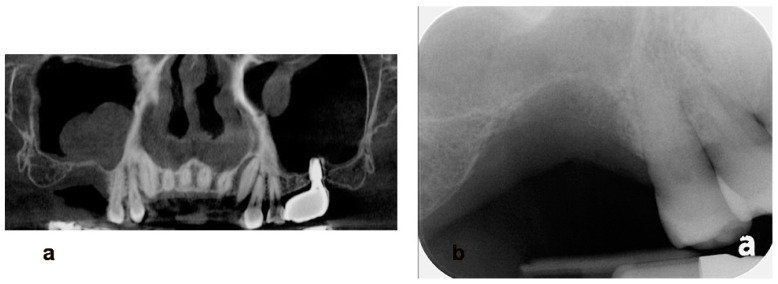
(**a**) Preoperative CBCT scans indicate a huge retention cyst on the right sinus. (**b**) A plain radiograph reveals 1 mm of residual bone height and an advanced ridge defect.

**Figure 11:**
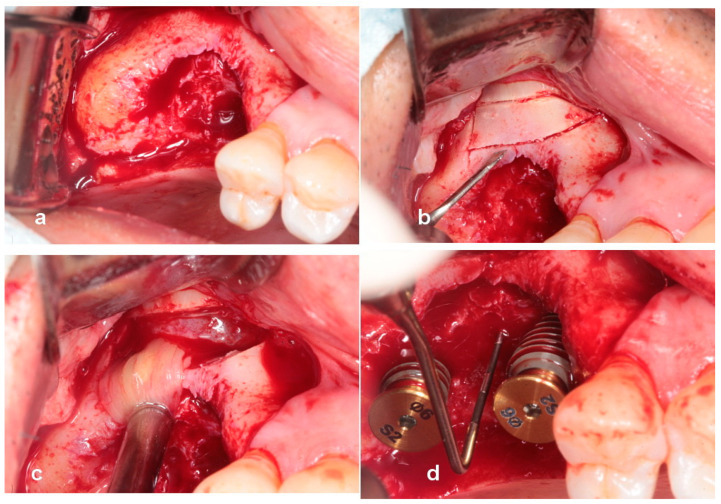
(**a**) Note the severe three-dimensional bone defect. (**b**) Replaceable osteoinductive bony window was prepared with a saw insert attached to piezoelectric bone surgery. (**c**) Cystic content was aspirated with a suction apparatus. (**d**) Implants were placed simultaneously and tenting pole abutment, as a vertical space maintainer, was placed on the implant platform, maintaining the bone graft over the exposed implant and bony defect. An approximately 10 mm vertical defect was revealed.

**Figure 12:**
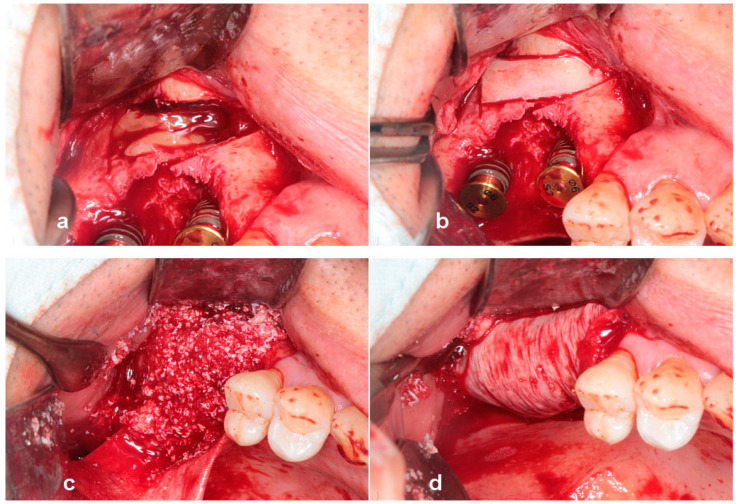
(**a**) Only CGF membranes were placed in the sinus to accelerate bone reformation in the sinus. (**b**) Replaceable osteoinductive bony window, as an osteoinductive barrier, was precisely repositioned in the lateral window. (**c**) Sticky bone was grafted over the defect. (**d**) A collagen barrier covered the bone graft without the placement of tacks.

**Figure 13:**
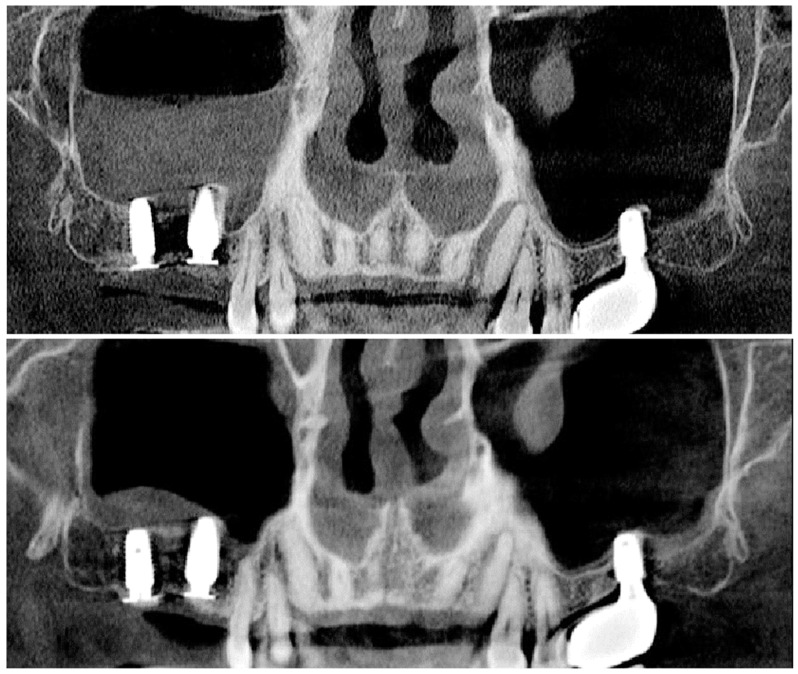
(**a**) Preoperative panoramic image of CBCT scan indicates mucosal elevation and bone grafting in the defect. (**b**) Panoramic image of CBCT scans after 5 months of healing reveals the resolution of a huge cyst and autologous bone reformation in the sinus. Successful ridge augmentation was also revealed.

**Figure 14:**
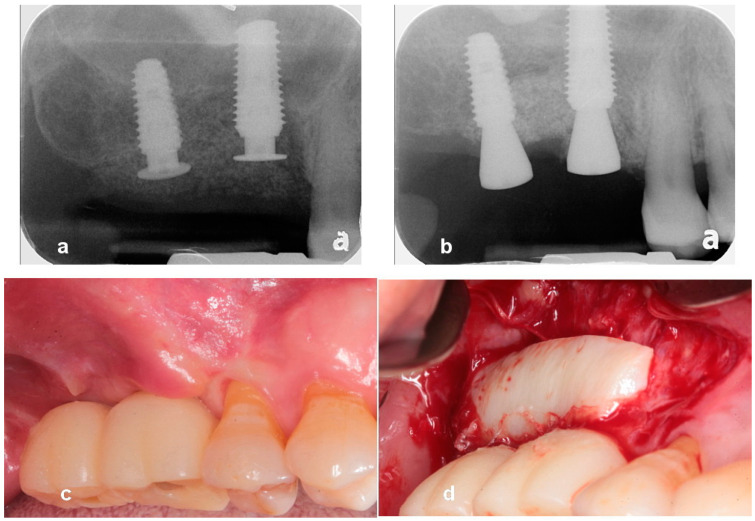
(**a**) A preoperative periapical radiograph. (**b**) A plain radiograph after uncovering procedure reveals an augmented ridge over implant platform. (**c**) Note the insufficiently attached keratinized gingiva around the implant restoration. (**d**) Suture-less free gingival graft was performed to widen the zone of the attached gingiva.

**Figure 15:**
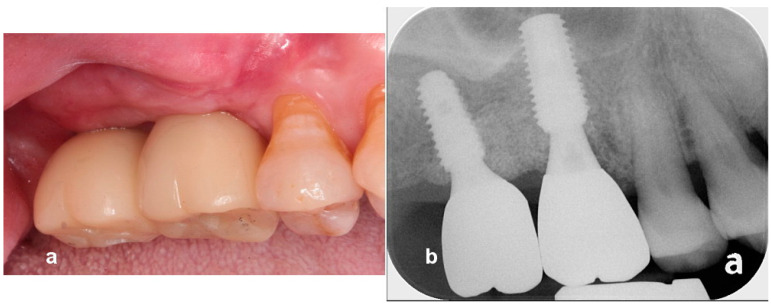
(**a**) An intraoral image after 6 months of loading reveals the attached keratinized gingiva around the zirconia-based final restoration. (**b**) A periapical radiograph after 6 months of loading indicates a stable augmented ridge over implant platforms.

**Case 3.** A healthy 58-year-old male was referred to our department for the reconstruction of a severely resorbed upper right posterior edentulous ridge. Preoperative CBCT image and plain radiograph revealed a huge retention cyst in the right maxillary sinus and severe three-dimensional defect with 1 mm of residual bone height in the edentulous ridge (Figure 10). Surgery was performed under local anesthesia through maxillary block anesthesia using 2% lidocaine that included 1:100,000 epinephrine. Flomoxef sodium (Flumarin^®^ Ildong pharmacentical Co., Seoul, 500 mg i.v., Republic of Korea) was administered one hour before surgery. A saw insert with a thin blade (S-Saw, Bukboo Dental Co., Daegu, Republic of Korea), connected to piezoelectric devices (Surgybone^®^, Silfradent srl, Sofia, Italy or Piezosurgery^®^, Mectron Co., Carasco, Italy), was used with copious saline irrigation to create the replaceable osteoinductive bony window. The anterior vertical osteotomy was made 2 mm distal to the anterior vertical wall of the maxillary sinus and the inferior osteotomy was prepared 2 mm above the sinus floor. The height of the vertical osteotomy was approximately 10 mm. The anterior and inferior osteotomy line were created perpendicular to the inside of the maxillary sinus lateral wall, and then superior and posterior osteotomies were made perpendicular to the sinus wall. This osteotomy design facilitates the precise repositioning of the bony window. The bony window was carefully detached to expose the sinus membrane after completion of the osteotomy in the lateral wall of the maxillary sinus. A stab incision with no. 15c blade was made on the exposed sinus mucosa, and a suction apparatus was inserted inside the sinus cavity to remove cystic content. After removing cystic content, elevation of the sinus membrane was continued until the medial wall of the maxillary sinus was exposed. Under-osteotomy was performed to place implants with favorable stability. A 5 mm wide × 10 mm long implant and a 6 mm wide × 10 mm long implant (Biotem implant, Seoul, Republic of Korea) were each placed as tenting pole screws so as to serve as space maintainers. Implants were placed 2 mm subcrestally to the adjacent proximal bone height. A tenting pole abutment with 2 mm high cuff (SANTA) was positioned on the implant platform as a vertical tenting device by which to allow for the over-grafting of the bone graft over the implant platform and was tightened to 10 Ncm. Six CGF membranes were placed in the new compartment under the elevated sinus mucosa to accelerate the healing of the perforated mucosa and bone regeneration in the sinus. The replaceable osteoinductive bony window was precisely repositioned in the lateral window as a bony barrier. The mixture of bovine bone (BONE-XBP, MedPark, Busan, Republic of Korea) and allograft (Accel, Ossgen, Daegu, Republic of Korea) was mixed with autologous fibrin glue to generate a sticky bone graft, as described in case report 1. The prepared sticky tooth bone graft was grafted on to the exposed implant surface and bony defect for three-dimensional ridge augmentation, a collagen membrane covered the bone graft, and tension-free suture was achieved. Tension-free sutures were created through the use of periosteal-releasing incision. Healing was uneventful before the uncovering procedure. Uncovering was performed after five months of healing, and CBCT scans indicated that favorable three-dimensional ridge augmentation over the implant platform was achieved. A progressive restoration was delivered two weeks after uncovering. The muscular pull from buccal mucosa around the implant-supported restoration was noted. A suture-less free gingival graft was performed to ensure the longevity of implant restoration. A final zirconia-based restoration was completed after two months of loading of the provisional restoration. After six months of loading, there was favorable maintenance of the augmented ridge (Figure 11, Figure 12, Figure 13, Figure 14 and Figure 15).

**Figure 16:**
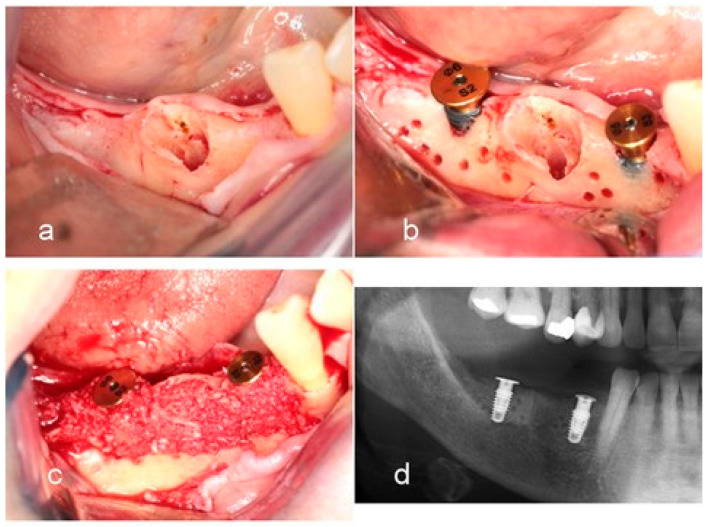
(**a**) There appeared to be a 3–4 mm vertical deficiency and a horizontal deficiency of up to 5 mm, requiring augmentation. The second premolar was electively extracted. (**b**) Tenting pole abutments were placed onto the abutments. Note that the versatile widths and heights of the SANTA abutments match the desired contour outcomes. (**c**) A sticky allograft was overlayed onto the buccal concavity. A collagen membrane was placed over the sticky bone graft and CGF membranes were overlayed. (**d**) Post-operative panoramic detail. Note the SANTA abutment rims stabilizing the graft material above the implant shoulders.

**Figure 17:**
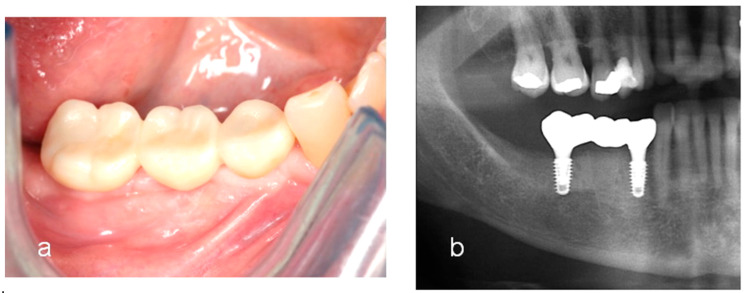
(**a**) Final screw retained implant bridge in place. (**b**) Note the presence of sufficiently thick and dense bony collars surrounding the implant shoulders, ensuring an excellent long-term prognosis and elimination of the risk of peri-implantitis.

**Case 4.** A 70-year-old healthy female was referred to our clinic for an assessment of dental implant therapy in the lower right mandible. The patient presented with a missing first bicuspid, an endodontically treated second bicuspid, and a recently extracted remaining molar. It was proposed that the lone bicuspid be extracted, and that two dental implants be placed to support a three-unit implant bridge. The recipient sites for the implants were deemed deficient in bony width and height and would require bone grafting for long-term success. To reduce morbidity for this elderly patient, it was proposed that the bicuspid extraction, implant placement and ridge augmentation be completed in a single simultaneous surgery. Under local anesthetic block and infiltrations (Bupivicaine 0.5% 1:200,000 epinephrine and lidocaine 2% 1:100,000 epinephrine), the flap design originates with a vertical remote release on the mesial of the canine, sparing the papilla. This is carried through around the canine and crestally around the bicuspid, terminating in a ‘hockey stick’ angle at the base of the ramus, approximating the external oblique ridge. Full flap dissection was achieved, with partial mental nerve dissection exposure as needed on the buccal and mirrored on the lingual with a small vertical release at the canine. The bicuspid root was atraumatically extracted using a Piezoelectric blade under copious sterile saline irrigation (Mectron, Piezosurgery Inc., Carasco, Italy). Two implants were placed, both 4.5 mm diameter × 10 mm length (MegaGen AnyOne, Daegu, Republic of Korea). Both implants were placed subcrestally (3 mm for the bicuspid implant, 2 mm for the molar implant) to the adjacent proximal bone height. Both implants achieved a primary torque of 40 Ncm. SANTA tenting pole abutments, as described in the previous cases, were placed on both implants (3 mm height/5 mm diameter for the bicuspid and 2 mm height/6 mm diameter for the molar) and seated at 20 Ncm. Buccal plate decortication was carried out electively. Horizontal periosteal releasing incisions were carefully scored on the mid-apical internal surfaces of both the buccal and lingual flaps using fresh no. 15c blades. This ensures adequate flap extension from both the buccal and lingual directions. Sticky allograft (OsteOss HansBiomed, Seoul, Republic of Korea) was overlayed onto the buccal and crestal aspects, as well as infilling of the bicuspid socket (Figure 16). A resorbable collagen membrane (Cytoplast, Osteogenics, Lubbock, TX, USA) was used to overlay the graft without tacks. CGF membranes were overlayed and tightly approximated, ensuring primary closure, using a combination of Monocryl and PTFE sutures. The panoramic detail shows that the SANTA abutments are level with the intended height of crestal regeneration. The uncovering was performed after six months of healing. Dimensional augmentation levels were successfully achieved, and a definitive restoration of the zirconia bases was delivered (Figure 17). 

## 3. Discussion

Various surgical techniques were utilized for the severe resorption of alveolar bone reconstruction. The use of an intraosseous or extraosseous bone block for autologous block bone grafting was regarded as the gold standard for reconstructing an atrophic alveolar ridge [18,19]. However, the grafting of intraoral and extraoral autoblock bone are known to cause complications, including wound dehiscence, hematoma, inflammation, fracture of the mandible, neurosensory disturbances, prolonged postoperative pain, significant morbidity, and a high risk of iliac bone fracture. Block bone grafts are known to have excellent space-maintaining capabilities, but high rates of unpredictable bone resorption after transplantation have been identified as a significant drawback [20,21,22].

Distraction osteogenesis can achieve vertical bone augmentation without the need for harvesting bone from other areas [23]. However, distraction osteogenesis can result in the improper control of force vectors and malpositioning of the distracted segment. This procedure has the disadvantage that it does not provide horizontal bone augmentation, necessitating additional horizontal bone grafting procedures [24].

The sandwich technique with interpositional bone graft allows for a vertical bone augmentation of 6–10 mm without the need for harvesting bone from other sites [10]. This technique utilizes a pedicle bone graft and has the advantage of minimal resorption over time compared with the use of a free autogenous bone block. However, it does not provide any horizontal ridge augmentation. The ramus split bone technique has been employed to address vertical bone deficiencies, as an alternative to block bone grafting [25]. The common disadvantage of the abovementioned techniques is that the implant cannot be placed simultaneously, which leads to a longer edentulous period for the patient during the healing phase and increased surgical visits. Additionally, these techniques are associated with a higher level of surgical complexity. It is known that guided bone regeneration has the advantage of being simpler and of causing less trauma than other complex bone augmentation surgeries. However, it is also known to have limitations in terms of vertical bone augmentation. Block bone grafting achieved a significant increase in vertical gain compared with the use of particulate material only when autogenous block grafts from extraoral donor sites were used [26]. In guided bone regeneration, non-resorbable membranes with excellent space-making ability were shown to result in greater vertical bone augmentation compared with resorbable membranes; however, these are also associated with an increased risk of membrane exposure [27,28]. Based on other research, it can be concluded that guided bone regeneration achieved a vertical bone gain with fewer complications compared with bone blocks in vertical bone regeneration. However, because guided bone regeneration has limitations in vertical augmentation, the guided bone regeneration technique was utilized with a tenting pole screw to provide more space maintenance and allow for the formation of new bone [29,30,31]. According to another study, tenting pole techniques, in conjunction with guided bone regeneration, revealed an average vertical bone gain effect of 9.7 mm, and all implants were integrated and successfully restored. After a mean follow-up of 16.8 months, the tenting of periosteum and soft tissue matrix with titanium screws is known to lead to a considerable and stable increase in alveolar ridge height and width [32,33]. However, the disadvantage of this technique is that it requires multiple surgeries and a longer edentulous period for the patient because implants cannot be placed simultaneously. In contrast, successful results have been obtained with the tenting pole technique using implants instead of screws in several studies [15,34,35]. This technique has the advantage of reducing the number of surgeries and shortening the edentulous period for the patient. The dimensional resorption that occurs over time in the augmented ridge due to remodeling cannot be avoided; therefore, over-grafting is necessary to compensate for this resorption [36,37,38]. A surgical technique using a tenting pole abutment has been introduced to provide space for bone grafting material to be placed on to the platform of the implant, for the purpose of tenting severely resorbed alveolar bone [16]. The tenting pole abutment technique with the guided bone regeneration procedure prevents the collapse of the space produced by the bone graft and minimizes the resorption of the grafting material in horizontal and vertical ridge augmentation procedures, as presented in this report. This technique is technically easier and has minor complications compared with other complex augmentation techniques, including block bone grafting or mesh-assisted ridge augmentation. In addition, this procedure shortens the edentulous healing period and reduces the frequency of required surgeries [39]. In order to achieve successful bone regeneration through this surgical technique, it is crucial to incorporate sticky bone to prevent the displacement of the particulate bone graft. When sticky bone is transplanted to the bone defect site, the transplanted material remains stable in the bone defect area. This eliminates the need to secure the barrier membrane in place with tacks during surgery, resulting in a shortened surgical time. In addition, sticky bone facilitates the slow release of growth factors, promoting tissue regeneration and reducing postoperative discomfort [17,40]. A properly executed tissue closure without tension is crucial at the surgical site. If the wound opens early and exposes the biomaterials to the oral cavity, it can lead to failure in bone regeneration. In this procedure, implant placement in the bone defect is essential. To ensure the initial stability of implants placed in the defect, at least 3mm of the apical portion of the implant must be engaged into the alveolar bone. Therefore, when employing this technique in the mandibular molar region, there should be a minimum of 5 mm of available alveolar bone superior to the inferior alveolar nerve to prevent damage to the inferior alveolar nerve during the procedure.

## 4. Conclusions

The successful clinical and radiographic results of the cases suggest that the utilization of tenting pole abutments connected to implants can lead to predictable outcomes, achieving three-dimensional ridge augmentation and preventing marginal bone resorption below implant platforms over time. Future investigations are required to validate the effectiveness of this technique.

## Data Availability

The datasets used in the report is available from the corresponding author on reasonable request.
